# A Novel Approach to Accounting for Loss to Follow-Up when Estimating the Relationship between CD4 Count at ART Initiation and Mortality

**DOI:** 10.1371/journal.pone.0069300

**Published:** 2013-07-30

**Authors:** Matthew Fox, Owen McCarthy, Mead Over

**Affiliations:** 1 Center for Global Health and Development, Boston University, Boston, Massachusetts, United States of America; 2 Department of Epidemiology, Boston University School of Public Health, Boston, Massachusetts, United States of America; 3 Health Economics and Epidemiology Research Office, Department of Medicine, Faculty of Health Sciences, University of the Witwatersrand, Johannesburg, South Africa; 4 Center for Global Development, Washington, District of Columbia, United States of America; The University of Tokyo, Japan

## Abstract

**Background:**

While CD4 strongly predicts mortality on antiretroviral therapy (ART), estimates from programmatic data suffer from incomplete patient outcomes.

**Methods:**

We conducted a pooled analysis of one-year mortality data on ART accounting for lost patients. We identified articles reporting one-year mortality by ART initiation CD4 count. We estimated the average mortality among those lost as the value that maximizes the fit of a regression of the natural log of mortality on the natural log of the imputed mean CD4 count in each band.

**Results:**

We found 20 studies representing 64,426 subjects and 51 CD4 observations. Without correcting for losses, one-year mortality was >4.8% for all CD4 counts <200 cells/mm^3^. When searching over different values for mortality among those lost, the best fitting model occurs at 60% mortality. In this model, those with a CD4≤200 had a one-year mortality above 8.7, while those with a CD4>500 had a one-year mortality <6.8%. Comparing those starting ART at 500 vs. 50, one-year mortality risk was reduced by 54% (6.8 vs. 12.5%). Regardless of CD4 count, mortality was substantially higher than when assuming no mortality among those lost, ranging from a 23–94% increase.

**Conclusions:**

Our best fitting regression estimates that every 10% increase in CD4 count at initiation is associated with a 2.8% decline in one-year mortality, including those lost. Our study supports the health benefits of higher thresholds for CD4 count initiation and suggests that reports of programmatic ART outcomes can and should adjust results for mortality among those lost.

## Introduction

The rapid global expansion of antiretroviral treatment (ART) programs has led to an increase in the number of people initiated on treatment worldwide to over 5 million by the end of 2010 [1]. While substantial work remains to be done, evidence from resource-limited settings shows clinical, immunological and survival outcomes have generally been favorable[2]–[9].

As policy makers and program managers evaluate ways to improve treatment outcomes, and assess the long-term costs and benefits of treatment,[10]–[17] it is important to be able to accurately assess the role of starting CD4 count in determining overall mortality on treatment. Starting CD4 counts continue to average roughly 100 cells/ml [3], [18] and low baseline and current CD4 count have been identified as major predictors of mortality on ART[4], [6], [19]–[21]. Still, these estimates have been made in the context of programs where ART program retention (i.e. alive and in care) has been sub-optimal [22]. While recent systematic reviews concluded two-year retention on ART was 60–70%, [22], [23] many patients who start treatment become lost to follow-up (LTF). Having large numbers of patients whose vital status is unknown makes estimating the impact of treatment programs challenging. A review of outcomes among lost patients [24] found about 40% of those lost from ART programs had died. While some patients who leave care will seek treatment elsewhere, for the remainder mortality is high and if not incorporated into assessments of the effectiveness of ART programs, the benefits of ART scale-up will be overestimated.

Several approaches to adjusting estimates of mortality for losses have been developed[25]–[30]. Each can be used to adjust program specific data to estimate program effectiveness. However, as each aims to adjust the results of a single program for losses, none utilizes all the published data on first-year mortality and loss to follow-up to derive comprehensive estimates of the association between loss adjusted mortality and baseline CD4 count.

To better characterize the potential relationship between increasing starting CD4 count and mortality while at the same time accounting for programmatic losses, we estimated the relationship between one-year on treatment mortality and baseline CD4 count, with and without adjusting for losses. We propose a new approach to estimating mortality among those lost as the best-fitting adjustment to the relationship between observed mortality and baseline CD4 count.

## Methods

### Study Selection

We sought to estimate the expected one-year mortality proportion as a function of initiating CD4 count using data from the literature. To identify potential studies for inclusion we searched PubMed, Medline, Web of Science, and Cochrane Reviews for all articles reporting one-year mortality by CD4 count at ART initiation (search terms shown in Appendix S1 in File S1, studies shown in Appendix S2 in File S1). We used data on patients initiating first-line three-drug combination ART in sub-Saharan Africa. We excluded studies which: 1) did not report the ART initiation CD4 count for patients on which mortality was calculated; 2) included cohorts from outside sub-Saharan Africa; or 3) were explicitly focused on special patient groups (e.g. children, tuberculosis patients). We included estimates of mortality by CD4 count even when incomplete follow-up on patients existed and used imputation (see below) to adjust mortality estimates for missing outcomes. Because we wanted observed estimates of mortality, we made no assessment of quality. When two reports using the same dataset were found (N = 6), we used the one with the larger sample size. Our search resulted in 20 unique studies representing 64,426 total subjects.

### Data Abstraction

We abstracted data on CD4 count, mortality and loss to follow-up from each study. While we sought to estimate the impact on one-year mortality of CD4 count as a continuous variable, most studies reported mortality by CD4 categories (e.g. <50, 50–100, etc.). For analysis, we considered each of the 51 CD4 categories from the 20 publications as unique observations. For each CD4 strata, we abstracted the upper and lower limits of the strata, one-year mortality, proportion of LTF and sample size. We found that roughly 80% of the data were from CD4 strata <200. If the stratum-specific sample sizes were not stated, they were imputed from the study’s total sample size (see below).

## Statistical Methods

### Regression Model

To estimate mortality among those lost we assumed that the most important determinant of mortality, including among those lost, was their CD4 count at ART initiation. Furthermore, we hypothesized that loss of patients masks a portion of total mortality, in such a way that the correlation between CD4 count at initiation and mortality appears weaker than if all mortality were observed. Our estimation strategy was to search for the value of mortality among those lost that maximizes the correlation between total mortality and CD4 at initiation. Our approach can be likened to a quasi-maximum likelihood estimation of the parameter *UM* (defined below) and our estimate of the parameter β is conditional on our estimate of *UM*.

To accommodate the pronounced non-linearity we observed in the relationship between mortality and CD4 at initiation, we postulated a log-linear relationship between unobserved total mortality,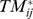, and median observed CD4 count at ART initiation, *CD4_ij_*. The hypothesized relationship is as follows:
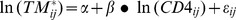
(1)where subscripts *i* and *j* respectively index the published study and the CD4 band within the study. The superscript * designates an unobserved variable. The coefficient β is is the slope parameter for the CD4 count and is expected to be negative. It can be interpreted as the percentage decline in total mortality associated on average with a percentage point increase in initiating CD4. The variable is a random variable, which we assume to be normally distributed and uncorrelated with *ln(CD4_ij_)* with variance proportional to the number of individuals initiating ART within CD4 band *j* of study *i*.

Unobserved total mortality in CD4 band *j* of study 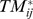, is a weighted average of observed and unobserved mortality, *OM_ij_* and 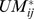, with the proportion lost in that band, *LTF_ij_*, as the weight:
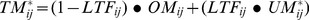
(2)where a superscript * again denotes an unobserved variable. As no study in our review reports LTF by CD4 at initiation, we approximated *LTF_ij_* by the overall study LTF, *LTF_i_*. We replaced the unobservable mortality among those lost in equation (2) by the parameter we wish to estimate, *UM*, defined as the proportion of those lost who die during the year, assumed constant across all CD4 bands and studies. Equation (2) then becomes:



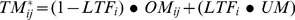
(3)To estimate *UM*, the unobserved mortality among the LTF, we substitute a value between 0–1 into equation (3) to yield estimated values of total mortality, 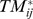. We use these estimated values of TM in place of the unobserved values on the left-hand-side of equation (1) and estimate the regression equation. We repeat this process until we find the value of *UM* yielding the best-fit.

Our main analysis assumes that the unobserved mortality among patients lost is constant across CD4 strata. In reality, mortality among those lost likely varies by CD4 count. Because the unobserved mortality amongst those lost may vary by CD4 count, we fit a second model in which unobserved mortality increased with decreasing CD4 count. In this analysis we relaxed the assumption of a constant mortality rate among those lost by changing the method of estimation of the unobserved mortality and replaced the single parameter value for unobserved mortality with a function that varies with starting CD4. Where we previously incremented mortality rates among those lost to follow up according to equation (1), we now treat unobserved mortality as a linear function as:
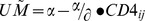



Where *α* is the unobserved mortality among those with a CD4 count of 0 and ∂ is the value of CD4 high enough for one-year mortality to be 0, i.e. 500 or above. We assumed that *α* = 0.85 since few patients who initiate treatment with a CD4 count of zero survive the year. We can then infer the unobserved mortality, *UM*, for any initial CD4 count in the data (CD4_ij_). We varied ∂ in 100 cell increments from 500–1000 which gives us six slopes. In other words, by specifying the mortality of those with a CD4 count of 0 (the y-intercept, set at 85%) and the CD4 where mortality is zero (the x = intercept varied between 500–1000), we have a set of six linear functions, each with a unique slope. The slope of the lines decrease as the × intercept increases, so the largest (i.e., the steepest fall in mortality among those LTF) has the x-intercept of 500. As before, we used 1000 imputations. In order to find the slope of the line, we iterated the × intercept of starting CD4 count from 500 to 1000 in steps of 100.

### Multiple Imputation

None of the included studies presents the median/mean CD4 count among the *n_ij_* patients in CD4 band *j* of study *i* but rather they typically define a CD4 band by lower and upper bounds. This requires us to select a value of CD4_ij_ to represent the CD4 count of the *n_ij_* patients in band *j* of study *i* before we can implement our estimation strategy. We address this problem with multiple imputation [31]. The complete estimation strategy, incorporating multiple imputation, consists of this sequence of steps shown in Table 1. We executed this estimation strategy twice, once using distributions for CD4 imputation informed by sample size and once uniformed by sample size (see Appendix S3 in File S1).

**Table 1:** Estimation strategy.

Step	Action
0	Set the value of unobserved mortality among LTF, *UM*, to zero.
1	Randomly select a value of *CD4_ij_* to represent the median CD4 count in CD4 band *j* of study *i*, for all *i* and *j*. Imputed values are drawn from a specified beta distributions defined over the interval between the lower and upper bounds of the CD4 band (or if no upper bound, the lower bound plus 100). We use left-skewed distribution used to impute the median CD4 count in the highest (open ended) CD4 strata, right-skewed distributions for bands with 0 as a lower bound, and symmetric distributions for interior bands (shown in Appendix S3 in File S1).
2	Randomly impute values of *LTF_i_* for two studies where LTFU was not reported.
3	Use equation (3) to compute the value of the dependent variable, ln(TM_ij_).
4	Estimate equation (2) by weighted ordinary least squares of ln(TM_ij_) on ln(CD4_ij_). To correct for heteroskedasticity, weight each observation by its sample size. For studies which did not report sample size by CD4 band (n = 5), randomly allocate total sample size across bands.
5	Repeat step 1–4 1,000 times
6	Combine the 1,000 estimated regression results to compute a pooled estimate of β, its standard error and the F-statistic measuring the goodness-of-fit of the regression. Apply Rubin’s formula[35]–[37] to estimate the pooled standard error of β: 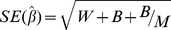 where W is the average within-imputation variance of 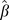 , and *B* is the variance of the estimator across *M = 1,000* imputations.
7	Repeat steps 1–6 10 times, incrementing the value of *UM* by 0.1 each time

## Results

**Figure 1:**
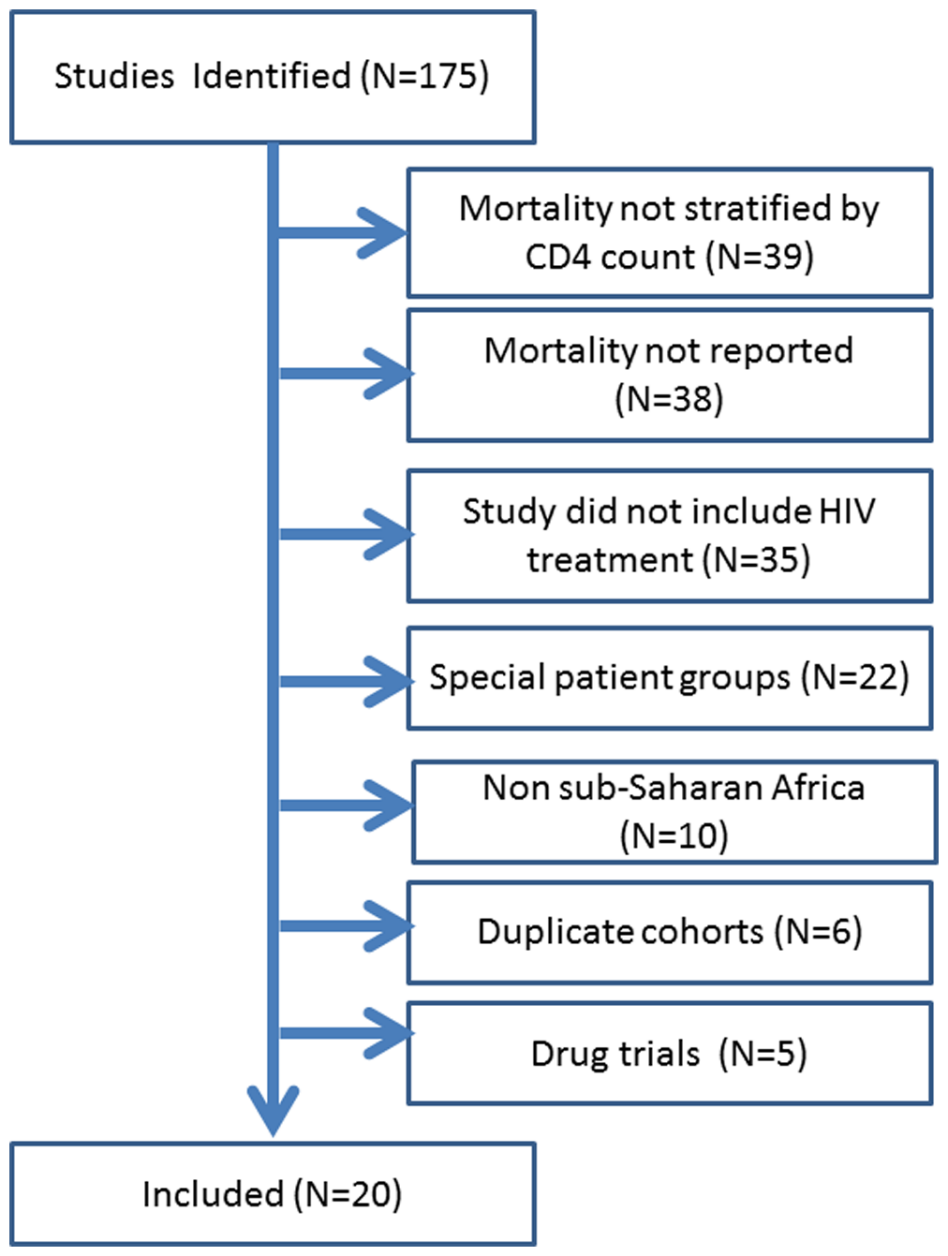
Flowchart of identified studies, included studies and reasons for exclusion.

We identified 175 studies, of which 20 met our inclusion criteria (Figure 1). Table 2 shows characteristics of the 20 studies which gave data on 65,336 patients from 10 countries and 3 multi-country studies. Of the single-country studies, 30% (5/17) were from Southern Africa. Reports ranged from a sample size of 101 to 11,776. The median starting CD4 count ranged from 65 to 150 cells/mm^3^. Overall reported mortality ranged from 1.3% to 18% while reported losses ranged from 0% to 32%.

**Table 2:** Characteristics of 20 studies included in the meta-analysis of the relationship between baseline CD4 count and first year mortality on antiretroviral therapy.

	Study, Year#	Country	N	MedianBaselineCD4*	Number ofCD4 strata	LowestCD4Strata	HighestCD4Strata	Total % Mortality	Total Loss to Follow up**	Reported Follow up Time (months)
**1**	Barth, 2008	South Africa	606	67	4	0–50	>200	18	15	12
**2**	Bisson, 2008	Botswana	410	81	0	N/A	N/A	8	21	11
**3**	Bourgeois, 2005	Cameroon	109	150	2	0–50	>50	8.3	2.8	16
**4**	Brinkhof, 2009	Multiple	10714	87–131	5	0–25	>200	8.8	7.1–31.7	24
**5**	Bussman, 2008	Botswana	633	67	2	0–50	>50	8.9	17.3	12
**6**	Calmy, 2006	Multiple	6961	89	5	0–15	>200	10	4.8	24
**7**	Cornell, 2009	South Africa	2023	102	4	0–50	>150	4.9	6.2	12
**8**	Culbert, 2007	Congo, D.R.	494	124	0	N/A	N/A	5.4	7.9	12
**9**	Etard, 2006	Senegal	404	128	3	0–50	>200	11.7	1.7	12
**10**	Geng, 2008	Uganda	3628	95	0	N/A	N/A	1.3	23	11
**11**	Lowrance, 2009	Rwanda	3194	141	0	N/A	N/A	4.6	4.9	12
**12**	Marazzi, 2008	Multiple	3456	166*	3	0–50	>500	7.5	1.2	12
**13**	Mzileni, 2008	South Africa	2380	–	4	0–50	151–200	7.8	14.1	30
**14**	Mutevedzi, 2010	South Africa	5179	116	0	N/A	N/A	3.7	10.9	12
**15**	Ojikutu, 2008	South Africa	298	65	4	0–20	>100	16	7.4	8
**16**	Seyler, 2003	Ivory Coast	101	135	2	0–50	>50	9.9	0	24
**17**	Stringer, 2006	Zambia	11776	143	4	0–50	>350	7.1	2	18
**18**	Toure, 2008	Ivory Coast	10211	123	4	0–50	>150	15	19	32
**19**	Weidle, 2002	Uganda	342	73	2	0–50	>50	16	24	12
**20**	Zachariah, 2006	Malawi	1507	123	2	0–50	>50	10.3	11.7	12

# Full citations given in Appendix S2 in File S1.

* Denotes mean not median.

**Figure 2:**
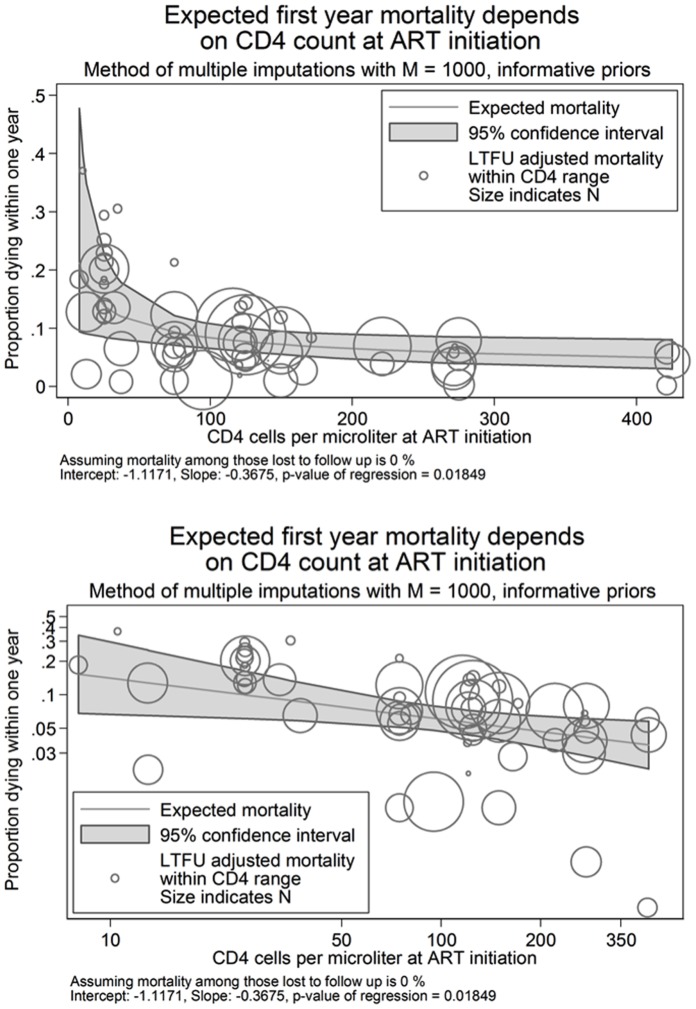
Predicted one year mortality by average imputed* CD4 count at antiretroviral therapy initiation assuming no mortality among those lost to follow-up with mortality expressed using a) an unlogged axis and b) a natural log axis. * 1000 imputations were used for each analysis.

After imputing CD4 values, we plotted CD4 against mortality (Figures 2a and b). The circles in Figure 2a show reported one-year mortality (unadjusted for loss) by the average imputed CD4 count with circle size proportional to the observation’s sample size. This figure shows that lower CD4 counts are associated with higher one-year mortality with the highest mortality associated with CD4 counts 0–50 cells/mm^3^. The decline in mortality with increasing CD4 is steep up to roughly a baseline CD4 count of 50 cells/mm^3^ and thereafter the reductions in mortality for starting ART at higher CD4 counts are reduced. The relationship is better illustrated in Figure 2b which plots mortality and CD4 count on the log scale and shows a roughly log-linear relationship between them. The estimated slope coefficient of −0.367 implies that a 10% increase in CD4 count at initiation is associated on average with about a 3.7% decline in observed mortality.

Predicted mortality by baseline CD4 count can also be seen in Table 3. Ignoring mortality among those lost and using distributions for imputing CD4 count uninformed by sample size (Table 3a, Row 1), predicted one-year mortality was highest among those with the lowest CD4 counts. Those with a CD4 count within the range at which most ART programs in resource-limited settings had been initiating patients onto ART without symptoms (*i.e.* ≤200 cells/mm^3^), one-year mortality was predicted to be above 3.5% at all values of baseline CD4 count below 500 and to increase sharply with lower CD4 count (*e.g.* those with baseline CD4 counts of 50 cells/mm^3^ had a 7.6% predicted one-year mortality risk). For patients initiated at CD4 counts ≥200 cells/mm^3^, mortality drops more slowly from a risk of 3.9% for a baseline CD4 count of 350 cells/mm^3^ (the threshold many programs have or are soon switching to) to 3.5% for a baseline CD4 count of 500 cells/mm^3^. Comparing those starting ART with a CD4 count of 500 cells/mm^3^ to those with CD4 count of 50 cells/mm^3^ the risk of one-year mortality was reduced by nearly 60% (3.5% vs. 7.6%) with an absolute risk reduction of 4.1%. Estimates using distributions for imputing CD4 count informed by sample size showed similar results (Table 3b, top row).

**Table 3:** Estimated mortality over the first year on antiretroviral therapy as a function of baseline CD4 count and assumptions about mortality among those lost to follow-up using beta distributions to impute CD4 counts that were a) uniformed by sample size and b) informed by sample size.

Estimated 1-year Mortality Proportion							
CD4 count at antiretroviral therapy initiation, cells/mm^3^							
Uninformed by sample size							
		1	50	150	200	350	500
Assumed mortality among those Lost to Follow up*	**0**	28.8	7.6	5.3	4.8	3.9	3.5
	**20**	29.9	9.7	7.1	6.5	5.6	5.0
	**40**	33.0	11.2	8.3	7.7	6.6	6.0
	**60**	35.7	12.5	9.4	8.7	7.5	6.8
	**80**	38.1	13.8	10.3	9.6	8.3	7.5
	**100**	40.4	14.9	11.3	10.5	9.1	8.3
**Informed by sample size**							
Assumed mortality among those Lost to Follow up*	**0**	32.7	7.8	5.2	4.7	3.8	3.3
	**20**	34.2	10.2	7.3	6.7	5.6	5.0
	**40**	37.9	12.0	8.7	8.0	6.8	6.1
	**60**	41.3	13.6	10.0	9.2	7.8	7.1
	**80**	44.4	15.1	11.2	10.3	8.8	8.0
	**100**	47.3	16.5	12.3	11.4	9.8	8.9

Each estimate of one-year mortality by baseline CD4 count presented so far ignores mortality among subjects lost (*i.e.* assumes 0% mortality among those lost), a scenario very unlikely to represent the true one-year mortality experience of all patients initiating ART. Figure 2b shows that while most observations fell close to our predicted line, several observations were far below it, partly because they do not account for mortality among those lost. We next fit a series of models assuming different values for mortality among those lost (ranging from 0 to 100% in increments of 10%) to identify the value of mortality among those lost which gave the best fitting model among the models we fit. As expected, overall one-year mortality (Table 3a and b) was lowest when assuming no mortality among those lost (*i.e.* naïve estimate) and highest when assuming 100% mortality among those lost. When assuming 100% mortality among those lost, overall one-year mortality was predicted to be above 14% for those starting ART with a CD4 count ≤50 cells/mm^3^, however this model did not provide the best fit to the data among the models we fit.

As our assumption about the amount of mortality among those lost increases, the model shows better fit (as summarized by the F-statistic in Appendix S4 in File S1) up to 60%, then the fit became somewhat worse. The best fitting model among those we fit occurred assuming mortality among those lost was 60% and estimates that, in the range of CD4 counts covered in the included studies, a 10 percent increase in CD4 count at initiation is associated on average with a 2.8 percent decline in overall mortality, including mortality among those lost to follow-up as well as those retained (Appendix S4 in File S1). These benefits to a higher initiation CD4 are larger than the 1.6 percent decline under the pessimistic assumption that all of those lost die during the first year.

**Figure 3:**
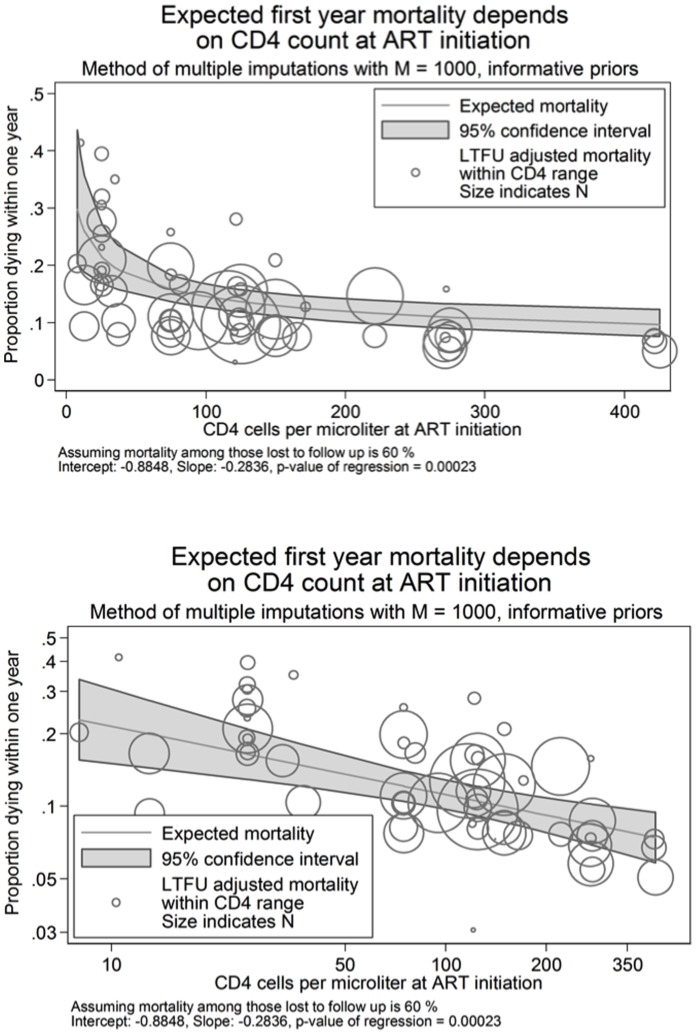
Predicted one year mortality by average imputed CD4 count* at antiretroviral therapy initiation assuming 60% mortality among those lost to follow-up with mortality expressed using a) an unlogged axis and b) a natural log axis. * 1000 imputations were used for each analysis.

Figures 3a and b and the fourth row of tables 3a and 3b show predicted one-year mortality assuming 60% mortality among those lost. Figure 3b shows that under this assumption, observations were much more tightly centered around the predicted values. The overall predicted line is shifted upwards and has a somewhat smaller downward slope (*i.e.* less reduction in mortality associated with increasing baseline CD4 count) compared with the naïve model in Figure 2b assuming 0% mortality among those lost.

Table 3a row 4 shows predicted one-year mortality by baseline CD4 count under the best fitting model assuming 60% mortality among those lost and distributions for imputing CD4 counts uninformed by sample size. Here, those with a baseline CD4 count ≤200 cells/mm^3^ had a predicted one-year mortality of roughly 8.7% or greater (e.g. 13.6% for those with a CD4 count of 50 cells/mm^3^) while those with a CD4 count ≥500 cells/mm^3^ had a one-year mortality risk ≤6.8%. Comparing those starting ART with a CD4 count of 500 to those at 50 cells/mm^3^ the one-year risk of death on ART was reduced by 54% (6.8 vs. 12.5%), less than when assuming no mortality among those lost, but with a greater reduction in absolute risk (5.7%). In this model, regardless of baseline CD4 count, mortality was estimated to be greater than in the naïve model assuming no mortality among those lost, ranging from a 23–94% increase (e.g. baseline CD4 count of 50 cells/mm^3^: 12.5% vs. 7.6% respectively and for a baseline CD4 count of 500 cells/mm^3^: 6.8% vs. 3.5% respectively).

**Figure 4:**
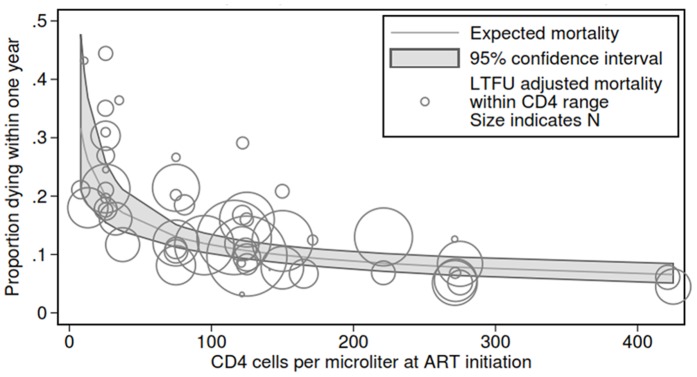
Predicted one year mortality by average imputed CD4 count at antiretroviral therapy initiation assuming a changing mortality among those lost to follow-up depending on CD4 count at ART initiation.

Our model in which we allowed unobserved mortality among patients lost is constant across CD4 strata is presented in Figure 4. Using this method, we obtained a vector of unobserved mortalities, rather than a single value, that varies linearly with starting CD4. All six of the slopes produce stable, significant coefficients of similar magnitude to our primary results. Figure 4 shows the expected mortality from our best fitting model. The results differed little from our model assuming constant mortality, with approximately 30% one-year mortality among those with a CD4 count approaching 0 with a sharp decline to roughly 10% mortality among those with a CD4 count between 100 and 150 at ART initiation.

## Discussion

This paper describes an approach to estimating the mortality experience over the first year on antiretroviral therapy as a function of baseline CD4 count. Our model which ignored loss to follow-up found that overall one-year mortality on ART in resource-limited settings was 7.6% among those initiated at CD4 counts of 50 and 3.5% in those initiated at a CD4 count of 500. Our findings are in line with a large analysis of data from sub-Saharan Africa by May and colleagues [32], but unlike previous analyses of this relationship, we also estimated predicted mortality among those lost and found that it adds substantially to observed mortality alone, across all starting CD4 counts.

Recently, WHO has recommended that resource-limited countries increase their initiating CD4 counts from 200 to 350 [33] and many countries are moving in this direction. Because to date so few programs have initiated patients onto ART at CD4 counts above 200 without a WHO stage III/IV condition, there is limited direct evidence from resource-limited settings to show the mortality benefits that could be achieved from increasing CD4 thresholds. [34], [35] Our findings demonstrate that patients initiated at lower CD4 counts are still at highly increased risk of death during the first year on ART compared to those initiated at higher CD4 counts and suggest that increasing CD4 thresholds could reduce overall mortality in ART programs.

Our study used data primarily from program evaluations of large-scale public-sector treatment scale-up. In such settings it is extremely difficult to determine the outcomes of patients lost, but it is likely that they experience substantial mortality [24]. While several methods have been proposed for adjusting program mortality rates for losses, many require diverting limited resources to patient tracing [28] In this study, we searched over a range of assumptions about mortality among those lost to determine which assumptions best fit the data on observed mortality and loss rates. After accounting for mortality among those lost, in our best fitting model we found that the one-year mortality proportion among patients initiated at a CD4 count of 50 increased to 12.5%, roughly double our estimates ignoring those LTF. Among those initiated at a CD4 count of 350, mortality increased from 3.9% to 7.5%. Thus our method is in agreement with others that ignoring mortality among those lost can substantially underestimate total mortality [30] and lead to an overly optimistic estimate of program success.

It is of note that when adjusting mortality estimates for losses we found that the assumption of 60% mortality among those lost provided the best fit to the data. A meta-analysis by Brinkhof [24] estimated that roughly 45% of those lost had died. While it is not clear from this analysis over what time period patients were lost in these studies and over what time period the mortality occurred, the finding, coupled with our finding, suggest that mortality among those lost is likely to be substantial. Given recent systematic reviews showing one year loss to follow-up to be between 20 and 25% percent [22] some adjustment should be attempted when reporting on programmatic treatment outcomes.

Our findings suggest that it is imperative that some adjustment be made to account for the impact of lost patients in our estimates of one year mortality. The results of this study suggest that a simple approach to gaining a rough guide to total mortality would be to assume 60% mortality among those lost. While such estimates could only be used as a guide, they could give program managers a sense for how much additional mortality they are missing.

Our study combined data from nearly 65,000 patients and was able to incorporate mortality both on treatment and among those no longer in care. However, our study has several limitations. First, our estimate of mortality by initiating CD4 count included data from subjects enrolled onto ART in resource-limited settings at CD4 counts well above 200. During the period of published data, few programs in these settings enroll such subjects into care without a WHO Stage III or IV condition. These patients may be sicker than the general population of patients who could be initiated at CD4 counts above 200. Thus, our mortality proportions for higher CD4 counts are likely overestimates of the actual mortality that would be experienced by this group and thus even greater gains in mortality may be achieved if well patients are initialed at higher CD4 counts as has been seen elsewhere [34]. Second, in our corrections for mortality among those lost we assumed that the mortality rate among those who dropped out was either constant or a simple linear function of initial CD4. However, it is more likely that CD4 count at the time of leaving care is a strong predictor of death [26]. Future efforts to adjust for LTF should account for the distribution of CD4 count at the time of leaving care. Third, it is important to note that there is the possibility that mortality rates differ by important site-specific characteristics which were not included in this analysis. These include the program location, the mix of services provided, and the mix of site personnel. Other sources of variation would include changes in the patient mix over time as programs mature and starting CD4 counts rise. Our results do not account for this variation. Finally we note that since roughly 80% of the data used from this analysis was from CD4 strata <200 this is the area where the strongest conclusion can be drawn.

In conclusion, in a large meta-analysis, we found a strong increased risk of death associated with initiating ART at lower CD4 counts over one year on ART in resource-limited settings. These risks increased substantially when accounting for mortality among those lost to follow-up. Our study supports increasing thresholds for CD4 count initiation and also suggests that reports of programmatic ART outcomes should attempt to adjust their results for mortality among those lost.

## Supporting Information

File S1 **File S1 includes Appendix S1, Appendix S2, Appendix S3, Appendix S4.** Appendix S1: Search terms used to identify studies of one year mortality on antiretroviral therapy. Appendix S2: Full citations for studies reviewed. Appendix S3: Illustration of a distribution used to impute CD4 count with bands. Appendix S4: CD4 coefficient (bottom) and model fit (F-statistic – top) for the relationship between one year mortality on ART and baseline CD4 count using varying assumptions about the amount of mortality among those lost to follow-up.
(DOCX)
