# Supplementary material for: A Novel Approach to Accounting for Loss to Follow-Up when Estimating the Relationship between CD4 Count at ART Initiation and Mortality
Source: PLoS One. 2013 Jul 30;8(7):e69300. doi: 10.1371/journal.pone.0069300 (PMC3728360; doi:10.1371/journal.pone.0069300)
Supplement: File S1 — includes Appendix S1, Appendix S2, Appendix S3, Appendix S4. Appendix S1: Search terms used to identify studies of one year mortality on antiretroviral therapy. Appendix S2: Full citations for studies reviewed. Appendix S3: Illustration of a distribution used to impute CD4 count with bands. Appendix S4: CD4 coefficient (bottom) and model fit (F-statistic – top) for the relationship between one year mortality on ART and baseline CD4 count using varying assumptions about the amount of mortality among those lost to follow-up. (DOCX) [file pone.0069300.s001.docx]

**Appendix 1 - Search terms used to identify studies of one year mortality on antiretroviral therapy**

| **Database** | **Search terms** |
| --- | --- |
| PubMed | ("humans"[MeSH Terms] AND ("HIV Infections/drug therapy"[Mesh] OR "HIV Infections/mortality"[Mesh]) AND "Africa"[MeSH Terms] AND ("lost to follow-up"[All Fields] OR "loss to follow-up"[All Fields] OR (losses[All Fields] AND follow-up[All Fields]) OR "late patients"[All Fields]) AND "CD4 Lymphocyte Count"[Mesh]) NOT "Clinical Trial "[Publication Type:noexp] |
|  | ("humans"[MeSH Terms] AND ("HIV Infections/drug therapy"[Mesh] OR "HIV Infections/mortality"[Mesh]) NOT ("Europe"[Mesh] OR "Australia"[Mesh] OR "north america"[MeSH Terms]) AND ("lost to follow-up"[All Fields] OR "loss to follow-up"[All Fields] OR (losses[All Fields] AND follow-up[All Fields]) OR "late patients"[All Fields] OR "dropout"[All Fields] OR "drop-out"[All Fields]) AND “CD4 Lymphocyte Count”[Mesh]) NOT "Clinical Trial "[Publication Type:NoExp] |
| Medline | (antiretroviral therapy and mortality and loss to follow up).mp. [mp=title, original title, abstract, name of substance word, subject heading word, unique identifier] |
| Web of Science | "antiretroviral therapy" AND "mortality" AND "loss to follow up" |
|  | Topic=(antiretroviral therapy) AND Topic=(mortality) AND Topic=(loss to follow up), Timespan=2000-2010. Databases=SCI-EXPANDED, CPCI-S. |
|  | TS=(haart OR art OR antiretroviral therapy) AND TS=(mortality) AND TS=(loss to follow up) AND TS=(initiation OR timing) AND TS=(Africa), 25 results Timespan=2000-2010. Databases=SCI-EXPANDED, CPCI-S. |
| Cochrane Reviews | (antiretroviral therapy and mortality and loss to follow up).mp. [mp=title, short title, abstract, full text, keywords, caption text] |

**Appendix 2 – Full citations for studies reviewed**

1. **Barth RE, van der Meer JTM, Hoepelman AIM, Schrooders PA, van de Vijver DA, Geelen SPM Tempelman HA (2008) Effectiveness of highly active antiretroviral therapy administered by general practitioners in rural South Africa. Eur J Clin Micro Inf Dis 27:977-984**
2. **Bisson GP, Gaolathe T, Gross R, Rollins C, Bellamy S, et al. (2008) Overestimates of survival after HAART: implications for global scale-up efforts. PLoS ONE 3:e1725.**
3. **Bourgeois A, Laurent C, Mougnutou R, Nkoue N, Lactuock B, et al. (2005) Field assessment of generic antiretroviral drugs: a prospective cohort study in Cameroon. Antiviral Therapy 10:335-341**
4. **Brinkhof MW, Boulle A, Weigel R, Messou E, Mathers C, et al. (2009) Mortality of HIV-infected patients starting antiretroviral therapy in sub-Saharan Africa: comparison with HIV-unrelated mortality. PLoS Med 6:e1000066**
5. **Bussmann H, Wester CW, Ndwapi N, Grundmann N, Gaolathe T, et al. (2008) Five-year outcomes of initial patients treated in Botswana's National Antiretroviral Treatment Program. AIDS 22: 2303-2311.**
6. **Calmy A, Pinoges L, Szumilin E, Zachariah R, Ford N, Ferradini L. (2006) Generic fixed-dose combination antiretroviral treatment in resource-poor settings: multicentric observational cohort. AIDS 20:1163-1169.**
7. **Cornell M, Myer L, Kaplan R, Bekker LG, Wood, R. (2009) The impact of gender and income on survival and retention in a South African antiretroviral therapy program. Trop Med Int Health 14:722-731**
8. **Culbert H, Tu D, O’brien DP, Ellman T, Mills C, et al. (2007) HIV treatment in a conflict setting; Outcomes and Experiences from Bukavu, Democratic Republic of Congo. PLoS Medicine 4:e129**
9. **Etard JF, Ndiaye I, Thierry-Mieg M, Gueye NFN, Gueye PM, et al. (2006) Mortality and causes of death in adults receiving highly active antiretroviral therapy in Senegal: a 7-year cohort study. AIDS 20: 1181-1189**
10. **Geng EH, Emenyonu N, Bwana MB, Glidden DV, Martin JN (2008) Sampling-based approach to determining outcomes of patients lost to follow-up in antiretroviral therapy scale-up programs in Africa. JAMA 300: 506-507.**
11. **Lowrance DW, Ndamage F, Kayirangwa E, Ndagije F, Lo W, et al. (2009) Adult clinical and immunologic outcomes of the national antiretroviral treatment program in Rwanda during 2004-2005.** J Acquir Immune Defic Syndr **52:49-55.**
12. **Marazzi MC, Liotta G, Germano P, Guidotti G, Altan AD, et al. (2008) Excessive Early Mortality in the First Year of Treatment in HIV Type 1-Infected Patients Inititiating Antiretroviral Therapy in Resource-Limited Settings. Aids Research and Human Retroviruses 24:555-560**
13. **Mutevedzi PC, Lessells RJ, Heller T, Barnighausen T, Cooke SG, Newell ML (2010) Scale-up of a decentralized HIV treatment programme in rural Kwazulu-Natal, South Africa: does rapid expansion affect patient outcomes? World Health Organ 88; 593-600**
14. **Mzileni MO, Longo-Mbenza B, Chephe TJ (2008) Mortality and causes of death in HIV-positive patients receiving antiretroviral therapy at Tshepang Clinic in Doctor George Mukhari Hospital. Pol Annals Int Med 118: 548-553**
15. **Ojikutu BO, Hui Z, Walensky RP, Zhigang L, Losina E, et al. (2008) Predictors of mortality in patients initiating antiretroviral therapy in Durban, South Africa. SAMJ 98:204-208**
16. **Severe P, Jean Juste MA, Ambroise A, Eliacin L, Marchand C, et al. (2010) Early versus Standard Antiretroviral Therapy for HIV-Infected Adults in Haiti. N Engl J Med 363:257-265.**
17. Seyler C, Anglaret X, Dakoury-Dogbo N, Messou E, Toure S, et al. (2003) Medium-term survival, morbidity and immunovirological evolution in HIV-infected adults receiving antiretroviral therapy, Abidjan, Cote d’Ivoire. Antiviral therapy 8: 385-393.
18. **Stringer JS, Zulu I, Levy J, Stringer EM, Mwango A, et al. (2006) Rapid scale-up of antiretroviral therapy at primary care sites in Zambia: feasibility and early outcomes. JAMA 296:7 82-793.**
19. **Toure S, Kouadio B, Seyler C, Traore M, Dakoury-Dogbo N, et al. (2008) Rapid scaling-up of antiretroviral therapy in 10000 adults in Cote d'Ivoire: 2-year outcomes and determinants. AIDS 22: 873-882.**
20. **Weidle PJ, Malamba S, Mwebaze R, Sozi C, Rukundo G, et al. (2002) Assessment of a pilot antiretroviral drug therapy programme in Uganda: patients’ response, survival, and drug resistance. Lancet 360:34-40.**
21. **Zachariah R, Fitzgerald M, Massquoi M, Pasulani O, Arnould L, et al. (2006) Risk factors for high early mortality in patients on antiretroviral treatment in a rural district of Malawi. AIDS 20:2355-2360.**

**Appendix 3 - Illustration of a distribution used to impute CD4 count with bands**

This figure shows an example of the distributions used to impute CD4 count, in this case a distribution for imputing a CD4 count from a band between 250 and 350 cells. In cases where we did not use sample size to inform the distribution, the distribution was very flat between its minimum and maximum value. When informed by sample size, the band gets narrower at its midpoint with greater sample size.

**Appendix 4 – CD4 coefficient (bottom) and model fit (F-statistic – top) for the relationship between one year mortality on ART and baseline CD4 count using varying assumptions about the amount of mortality among those lost to follow-up**
